# Potential Clinical Role of Telomere Length in Human Glioblastoma

**Published:** 2011-10-17

**Authors:** D. La Torre, M. Aguennouz, A. Conti, M. Giusa, G. Raffa, R. V. Abbritti, A. Germano’, F. F. Angileri

**Affiliations:** Department of Neurosciences, Psychiatric and Anesthesiological Sciences, University of Messina School of Medicine, Messina, Italy

**Keywords:** Telomere length, Glioblastoma multiforme, Glioma, Brain tumor, prognostic marker

## Abstract

Glioblastoma Multiforme (GBM) is the most common and lethal of human primary central nervous system (CNS) tumors. Due to the tumour’s intrinsic clinical and molecular heterogeneity, choice of initial treatment, prediction of survival, stratification of patients, prediction and monitoring of response to therapy, represent some of the greatest challenges in the management of GBM patients. Patients, despite optimal surgery, radiation and chemotherapy, still have a median survival of 14–16 months. A reason for this dismal prognosis is because of the relative inaccuracy of current prognostic markers, so far based on clinical or pathological variables. Molecular markers that effectively predict response to therapy and survival outcomes are limited. Consequently, there is a strong need to develop novel and independent markers of prognosis. Ideal biomarkers for solid tumors would serve one or more important functions. Telomeres, guanine-rich tandem DNA repeats of the chromosomal end, provide chromosomal stability, regulates important cellular processes, and seem to be implicated in human carcinogenesis. Recently, telomeres have been shown either to be associated with clinical markers of disease progression or to be independent markers of cancer prognosis in solid tumours, including GBM. Nevertheless, a corresponding comprehensive discussion of these promising developments in brain tumours has not yet been available in the literature. Therefore, here we reviewed studies focused on the assessment of telomeric length in brain tumours with the aim to emphasized those findings indicating a potential clinical role of telomeres in GBM. With the aim to enhance the awareness of the potential clinical role of telomeres’ length information in GBM, using a southern blot analysis, telomeric length in excised tumour samples was analyzed. Moreover, an attempt to correlated telomere length with patients’ overall survival, was also performed. The findings here reviewed shows some contradictory results, due to different tissues used as controls, but mainly to cellular and molecular heterogeneity in GBMs that drive molecular mechanisms controlling telomere length, included telomerase and Alternative Lengthening of Telomeres (ALT), through multiple mechanisms. However, overall these studies, including our own, are consistent with the hypothesis that GBMs’ telomeres were always shorter when compared with Normal Brain Tissue (NBT), and together with higher telomerase activity seem to be associated with malignancy and poor outcome; while tumours with ALT phenotype have longer telomeres, “*less malignant*” behaviour and better prognosis. We conclude that, although not entirely consistent in the type of telomere alteration, i.e., attrition vs. elongation, and unclear on the underlying mechanisms, multiple studies in brain tumours have shown that telomere dysfunctions are associated with parameters of clinical outcome in patients with GBMs and therefore will be part of novel risk assessment and prognostic modalities for patients with these still dismal disease.

## INTRODUCTION

Gliomas are a heterogeneous group of malignancies with highly variable outcomes. Pathological diagnosis is largely based on the histological appearance of the tumours. Despite notable recent achievements in oncology, malignant gliomas such as glioblastoma multiforme (GBM) present some of the greatest challenges in the management of cancer patients worldwide. Glioblastoma is the most common primary brain tumour in humans (1) and has the most severe prognosis (2, 3). Even with aggressive surgical resections using state-of-the-art preoperative and intraoperative neuroimaging, along with recent advances in radiotherapy and chemotherapy, the prognosis for GBM patients remains dismal: median survival after diagnosis is about 15 months (4). So far, the most useful prognostic tools in this disease remain clinical indices such as age, the Karnofsky Performance Status (KPS) score and the histopathologic grade, as was reported in the recursive partitioning analysis performed by the Radiation Therapy Oncology Group (RTOG) (5), as well as the Ki-67 proliferative index. However, the heterogeneity of gliomas has made prognostic determinations, based purely on clinicopathologic variables, difficult. Recently, attempts to categorize GBM according to the response to chemotherapy were proposed. Inactivity of the O6-methylguanine-DNA methyltransferase seems to increase the responsiveness of GBMs to alkylating agents. However, the O6-methylguanine-DNA methyltransferase promoter is methylated in only 45% of GBM (4, 6). EGFR and p53 are molecular markers that show promise as prognostic indicators of recurrence-free and overall survival in patients with GBMs, but further prospective studies are needed to confirm the retrospective findings. Postsurgical evaluation of these markers is potentially helpful in planning follow-up and treatment for these patients. Tumours expressing relatively high levels of these markers require closer follow-up and, when possible, more aggressive therapies. Despite intensive investigation into the expression of molecules regulating apoptosis in brain tumours, no evidence presently exists to support their usefulness as markers of patient outcome (7). Although GBM is one of the best-studied brain tumor in terms of genetics and molecular prognostic factors, the true prognostic significance of all potential factors under investigation remain to be clarified. Therefore, the absence of reliable biological markers allowing the assessment of the evolution and prognosis of these tumours remains a major impediment to the clinical management of those patients. It has recently been speculated that changes in telomere domain can result in genetic disorders, genomic variability and, cell immortalization (8). Telomeres consist of long tandem arrays of TTAGGG repeats bound by proteins collectively termed the shelterin complex, placed at the end of linear chromosomes, which are involved in several essential biological functions (9, 10). Functional telomeres protect chromosome ends from recombination and fusion, and are therefore essential for maintenance of chromosomal stability (9, 11, 12). Telomere dysfunction occurs as a result of critical shortening of telomeres, followed by sequential bridge–fusion–breakage cycles, leading to numerical chromosomal abnormalities (13, 14). Chromosomal instability is a phenotype that induces widespread genetic alterations, which may play critical roles in human carcinogenesis. The phenomenon of telomere alteration during tumorigenesis process and progression of solid tumors is well known and established at the molecular level. Cells exhibiting telomere dysfunction, with critical shortening and genomic instability, increase in both the formation of dicentric chromosomes and susceptibility to oncogenic transformation (15, 16, 17). Telomerase is a complex of a reverse transcriptase protein encoded by the TERT (telomerase reverse transcriptase) gene and a template RNA TERC (telomerase RNA component). Telomerase can add telomeric repeats onto the chromosome ends, and prevents the replication-dependent loss of telomere and cellular senescence in highly proliferative cells of the germline and in the majority of cancers (18). Thus, telomerase activity and telomere maintenance are associated with the immortality of cancer cells, germ-line cells, and embryonic stem (ES) cells (19). The presence of telomerase RNA or telomerase activity appears to correlate with degree of malignancy in multiple types of brain tumors, including gliomas; however, it currently has no use as an independent prognostic indicator of patient outcome (7). It may instead be a marker for malignant tumor initiation or progression (7). Instead, the relationship between telomere maintenance, genomic instability and the resulting phenotypic variability that gives rise to cell clones that cause disease recurrence is well accepted (20, 21). Also, the potential clinical use of telomere length information for the prognosis of solid tumours has been recognized and continues to be validated (22, 23, 24, 25, 26, 27, 28, 29, 30, 31, 32, 33). Here, we analyzed telomeres length in 15 glioblastoma samples obtained *in vivo*. Moreover, an attempt to correlate telomere length with patients’ survival was made. Finally, to increase our awareness of the potential clinical role of telomeres’ length information in glioblastoma, we reviewed, on the light of the recent literature, studies indicating the prognostic potential of telomeres in GBM. Excluded from this discussion studies focused on the use of telomerase, the enzyme implicated in telomere maintenance, as a biomarker for diagnosis or prognosis in brain tumours, a research area that also has been previously reviewed (34). The purpose of our study was to better define possible clinical significance of telomere dysfunctions in this disease.

## MATERIALS AND METHODS

### Patient population

This study included tumors samples, histologically verified as GBMs, obtained in adult patients who underwent craniotomy for microsurgical tumor resection, at the Department of Neurosurgery of the University of Messina. All tumors were located in the supratentorial compartment. Only patients who had undergone large, gross total resection of their neoplasms (more than 95 % of the tumor volume) were eligible for the study. All patients underwent Temozolomide chemotherapy (75 mg/m(2)/d x 7 d/wk for 6 weeks) administered orally concomitant with fractionated radiotherapy (60 Gy total dose: 2 Gy x 5 d/wk for 6 weeks) followed by temozolomide monotherapy (200 mg/m(2)/d x 5 days, every 28 days for six cycles). Samples obtained from single or multiple stereotactic biopsies were not included in the present study. We carefully excluded tumors containing components that were suspicious of oligodendroglioma. No case of recurrent tumors and no patient who underwent adjuvant therapy (radio and/or chemotherapy) before surgery were employed in the present study. Study included 9 men and 6 women whose mean age was of 66.66 ± 4.7 years, Karnofsky Performance Status (KPS) at the admission of 88.6 ± 9.1 and a mean survival period of 63.7 ± 17.3 weeks.

### Tissue Samples

All tumor tissue samples were obtained from resection specimens, within 15 minutes from surgical tissue removal. Specimens were taken from viable areas of tumor, avoiding areas of gross necrosis and three to seven anatomically separate areas of tumor tissue were sampled from each resection specimen, according to the volume of excised tissue available. Tissue samples for the histological, immunohistochemical, and telomere length analysis were taken from the same general region of the tumor during surgery. Tissue was placed in cryovials and immediately flash-frozen in liquid nitrogen in the operating room and stored at – 70° C. Both the adjacent tissue samples matched to the frozen tissue, as well as additional tissue submitted *in toto* from the resection specimens, were used for histological typing and grading.

As controls three samples of normal brain tissue (NBT) were used. Non-neoplastic brain tissue samples were derived from the temporal lobes of patients surgically treated for temporal lobe epilepsy and included histologically verified normal cortex and white matter.

### Telomere length analysis

Genomic DNA was extracted from tumour specimens using standard method phenol/chloroform. Terminal Restriction Fragments (TRF) mean length measurement was performed using Telo TTAGGG Telomere Length Assay kit (Roche Molecular Biochemicals, Mannhein, Germany) as previously described (35). Briefly, genomic DNA (2 μg) was digested with *Hinf*I and *Rsa*I, separated by agarose gel electrophoresis, transferred to a nylon membrane, and hybridized with a digoxigenin-labeled telomere-specific probe. Following to analysis, the TRF length was determined as the ratio of the length of tumor tissue TRF and their paired normal tissue TRF (ratio T/N). All cases in which T/N ratio resulted <1 were considered into the group of telomere shortening. When T/N was ≥1, tumors were considered in the group of telomere maintenance.

### Statistical analysis

The Pearson test was used to obtain correlation values among numerical variables. Data analysis was performed with INSTAT, v. 3.0, and PRISM, v. 4.0 (GraphPad, San Diego, Calif). A probability value less than .05 was considered statistically significant. All values are expressed as the means ± SD.

## RESULTS

Patients’ clinical and molecular parameters are summarized in ***Table 1***. Telomere length was measured by Southern blot analysis in primary, untreated glioblastoma samples and in matched normal tissue TRF (ratio T/N) from 5 patients. Telomeres length was determined as the ratio of the length of tumor tissue TRF and their paired normal tissue TRF (ratio T/N). All cases in which T/N ratio resulted <1 were considered into the group of telomere shortening. When T/N was ≥1, tumors were considered in the group of telomere maintenance. Changes in telomere length, compared with their paired normal brain tissue, were observed in all tumors; were shorter in 13 of 15 tumours (86.66%). While telomeric length in 2 tumors (13.33%), was considered unchanged. The average telomere lengths in tumor samples set were lower compared with the normal brain tissues. In the present study the average telomere length was 8.66 ± 2.34 Kbs in GBMs; while was 10.93± 2.35 Kbs in NBT. To define possible clinical significance of above mentioned findings, telomeres’ length was correlated with patients’ survival. Using a linear regression analysis an inverse correlation between telomere length and patients’ survival was found (P < 0.01). (***Table 1***)

## DISCUSSION

In the present study telomeric length in excised glioblastoma samples was analysed with the aim to contribute to the understanding of the role of telomere maintenance in brain tumours. Moreover, a systematic review of literature focused on the assessment of telomeric length in GBMs was performed. Our results demonstrate that telomere length varied among GBM samples but was always shorter as compared with normal brain tissues. The average telomere length was 8.66 ± 2.34 Kbs in GBM samples; while was 10.93± 2.35 Kbs in NBT. We also demonstrated an inverse correlation between telomere length and patients’ overall survival, suggesting a possible clinical role of telomere maintenance in glioblastoma. Our findings are in agreement with many authors studying telomeric length in different solid tumors, including breast, lung, colorectal, and head and neck cancers. Most of these studies suggest that in neoplastic tissue, telomeres are shorter when compared with normal tissue (22, 23, 24, 25, 26, 28, 29, 32, 33) and thus confirming the hypothesis that telomere shortening may represent an early event in carcinogenesis. It is noteworthy that in almost all these studies telomere attrition is also associated with parameters of increased risk and poor outcome in human cancers. In the present studies, telomere attrition was observed in 13 of 15 tumours (86.66%). While, in 2 tumours (13.33%), telomere length was considered unchanged. Noteworthy, the latter were those patients with worse overall survival. Therefore, we compare telomere length and patients survival to verify possible correlation between telomere length and survival in GBM patients. Using a linear regression analysis an inverse correlation between telomere length and patients’ survival was found (P < 0.01). These findings may suggest that relative increase of telomere length in a subset of GBM patients was associated with worse prognosis. Our findings are in concert with those reported for colorectal and lung cancer, in which increased TRF correlated with higher tumour stage, and decreased overall survival; but are in contrast to other studies reporting as reduced TRF was correlated with advanced stages in different cancer type, including breast and prostate cancers. One explanation for this discrepancy might lie in a potential tissue-specific pattern of telomere dysfunction that may reflect the underlying biology of telomere maintenance and its alteration over time in specific tissues. The latter is, for example, the case for normal colorectal epithelium that contain telomerase-positive cells of possible stem cell origin and presumably counteract telomere attrition due to physiologically high cell proliferation rates, and total cell loss due to physiological shedding in this specialized cell compartment (36). Thus it is possible that chromosomal instability and genetic disorders, due to telomere dysfunction, may affect the molecular key players of telomere maintenance, resulting in elongated telomeres in tumours. Telomeric DNA consists of short guanine-rich repeat sequences in all eukaryotes with linear chromosomes, and its length in human somatic cells is remarkably heterogeneous among individuals ranging from 5 to 20 kb, according to age, organ, and the proliferative history of each cell (9). During a process of DNA synthesis and cell division, telomeres shorten as a result of the incomplete replication of linear chromosomes, the so-called ‘end-replication problem’. To prevent degradation by exonucleases or processing as damaged DNA, the telomere 30 single-strand overhang folds back into the D-loop of duplex telomeric DNA to form a protective ‘T-loop’, which is reinforced with TRF2 and other telomeric DNA-binding proteins named shelterin (37). Telomerase is a complex of a reverse transcriptase protein encoded by the TERT (telomerase reverse transcriptase) gene and a template RNA TERC (telomerase RNA component). Telomerase can add telomeric repeats onto the chromosome ends, and prevents the replication-dependent loss of telomere and cellular senescence in highly proliferative cells of the germline and cancer cells. However, some tumour cells do not express telomerase activity; the stabilization of telomeres is maintained by telomerase independent mechanism, an ‘alternative lengthening of telomeres’ (ALT), when the G-strand overhang invade the loop structure and act as the template for its elongation (38, 39). Hence, even if most human cancers use telomerase as their telomere maintenance mechanism, some use an alternative lengthening of telomeres (ALT) mechanism. The latter especially occurs most often in tumors with complex karyotypes, astrocytic brain tumours and osteosarcomas. Therefore, at least two mechanisms of telomere maintenance, telomerase activity and the recombination- based ALT, may be more or less prevalent in different tissues undergoing tumour formation, leading to the observed differences (40, 41). In human tumours a hypervariability of telomere length was observed (42, 43, 44), with only few studies conducted to analyze telomere length variations in brain tumors (44, 45, 46, 47, 48). However, determination of telomere length variations in cancers may provide promising information for its potential role as prognostic marker in cancers. To completely ascertain this hypothesis, a better understanding of the molecular events underlying telomere alterations in tumours and histologically normal tissues adjacent to tumours should be analyzed.

### Literature review

Analysis of telomere length and its potential use as prognostic tool in GBM has been reported in several studies. ***(Table 2)***, but none was specifically addressed to clarify the role of telomere length as prognostic marker in GBM. Therefore, here we reviewed studies focused on telomere length in brain tumours with the aim to emphasize those findings useful to enhance the awareness of potential clinical role of telomeres in GBM. In 1993, Nunberg et al., first reported an analysis of telomere length in 60 intracranial tumor samples, including GBM. Probing with the 32P-labelled synthetic (TTAGGG) 3’oligonucleotide revealed length changes of the telomeres occurring in intracranial tumours. Among 60 samples analysed, 41.7% showed telomere elongation, and 21.7% telomere reduction, whereas 36.7% of the tumours exhibited equal lengths compared with the patients’ peripheral blood leukocytes (PPBL) (44). Years later, Liu J et al. analyzed TRFs in a series of 12 GBMs and different grade of astrocytomas compared to normal brain tissue. Using a Southern blot analysis, they observed a progressive shortening of TRFs in astrocytomas from WHO grade I to IV with any significant differences between primary and recurrent GBMs. These findings support the hypothesis that in GBM cells, the telomere repair mechanisms maintain a relative stability of TRFs and permit a constitutive proliferation of those malignant cells, confirming a possible clinical role of telomere length in GBMs (48). *Morii and colleagues*, analyzed telomere length and telomerase activity in a series of 20 gliomas (WHO grade I to IV), including 11 GBMs. In their series, 35% of the glioma samples examined were telomerase-negative. The authors demonstrated that telomerase-negative gliomas had longer TRFs compared with telomerase positive ones, suggesting that, in addition to the telomerase-dependent mechanism, a telomerase-independent mechanism for telomere maintenance may be present in human gliomas (47). The first report describing the relationship among ALT pattern, telomere length, and prognosis in human GBMs, was published by a group led by *Hakin-Smith et al*.. who analyzed telomerase activity and telomere lengths in 77 GBM patients. In their study, nineteen (25%) of 77 tumors presented telomeres longer than 17 kb and features of alternative telomere lengthening (ALT phenotype) on Southern blot. ALT phenotype patients had a median survival of 542 days (95% CI 114–970) compared with 247 days (224–270) in those without the ALT phenotype. In patients with non-ALT tumours, telomerase activity did not affect survival (median 287 [199–375] vs 236 [230–242] days, p=0·275). These findings suggest that ALT is associated with elongated telomeres, benign biology and better prognosis in GBMs (27) while telomerase activity did not correlate with survival. Other studies were focused on the relationship between telomerase activity and telomere length in brain tumors (34, 42, 49, 50). Particularly, *Hiraga et al*. published a wide series of 160 tumor samples including 47 GBMs, demonstrating that tumors with high telomerase activity presented very short telomeres. In contrast, telomerase negative samples had TRF lengths compatible with normal values (34). *Harada and colleagues*, confirmed this hypothesis and analyzed possible differences between primary and secondary GBMs. Summarizing their findings, telomeres in tumor samples were always shorter compared with NBT, but not statistically significant differences between primary and secondary GBMs were found. Interestingly, nevertheless the latter presented significantly higher levels of telomerase activity and hTERT expression than the former, telomere length was anyways shorter than normal brain tissue. The authors explained this apparent discrepancy suggesting that telomerase activation occurs late in carcinogenesis, when the high replication rate of tumor cells already caused the telomeres shortening. At this point, activation of telomerase represents the principal mechanism to escape to apoptosis and cell death. Conversely, in primary GBMs, shorter telomere length can be explained by a reduced telomerase activity that might have less influence on carcinogenesis and, hence, other unknown factors might facilitate their cellular immortality (50). The clinical role of telomere length or its proxies in brain tumors as marker of malignant potential has been specifically addressed just in several studies in the groups led by *Maes, Hiraga, and Le.* In a study encompassing 14 patients with different grade astrocytomas, including 11 GMB, *Maes et al,* determined that telomere length, compared with controls, was reduced in the high-grade tumours, such as GBMs, while was unchanged in the low-grade astrocytomas, suggesting that telomerase activity and hTERT, together with the telomere length can be an index of malignancy itself in intracranial tumors (49). Similar results were reported by others investigators who reported that telomerase activity is frequently detected in malignant astrocytic tumors, including GBMs, and it is associated to shorter telomeres (34, 42). These results suggested that telomerase activity and shorter telomeres detected in the more malignant stages of tumor progression can be useful as markers for malignancy of human gliomas. Finally, *Rahman et al.* compare telomere length with both constitutional blood and tumours of neuroectodermal origin. In their paper, Authors, demonstrated that the mean telomere length from tumors of of glial origin is significantly longer compared with neuroectodermal tumors and did not differ significantly from the mean blood telomere length from four patients (51). One of main difficulties to assess the role of telomere in GBM depend on marked heterogeneity at cellular and molecular levels of this neoplasm. Increasing evidences from the literature support the concept that a subpopulation of cancer cells in each tumor has greater potential of cancer initiation and repopulation (52, 53, 54, 55, 56, 57, 58, 59, 60, 61, 62). These cells are called cancer stem cells (CSCs) or tumor-initiating or propagating cells because they share some critical characteristics with normal stem cells, including the capacities for self-renewal, multi-lineage differentiation, and maintained proliferation (63, 64, 65, 66, 67, 68). For this reason, recently, several authors focused their interest to the analysis of different pattern of telomere length and telomerase activity within GBMs tumor initiating cells (TICs). According to their specific biological characteristics, different from that of the other bulk tumor cells, they represent a little tumor cell subpopulation that is thought to be responsible of the tumor progression. In a recent study, *Castelo-Branco et al*. suggested that telomerase-dependent telomere length maintenance in high grade gliomas and neuroblastomas might be critical only to the survival of the self-renewing TIC tumor subpopulation and not to the bulk of tumor cells or the corresponding tissue stem cells. Authors demonstrated that TICs from GBM and NBL samples, possess high levels of hTERT and telomerase activity, accompanied by very short telomeres. Conversely, the majority of bulk tumor cells, normal neural stem cells and neural crest–like stem cells, have longer telomeres and undetectable telomerase activity (probably due to low hTERT expression) (69). These data support the hypothesis that tumors, including GBMs, are composed of a heterogeneous group of cells with different telomere maintenance. These findings are consistent with those of *Marian et al.* who reported that telomere length in GBM tumor cells and in GBM TICs were shorter (∼3.5kb), than normal brain cells telomeres. Furthermore, this study clearly show that the average telomere lengths of GBM tumor cells are approximately three times shorter compared to normal human brain cells (∼3.5 kb vs. ∼10) (70). According to the literature, the presence of shorter telomeres suggests a late activation of telomerase during the carcinogenesis by all tumor cells that can escape crisis. In these cells, telomere length is initially short and, in a late stage, following telomerase activation, tend to become longer. This mechanism seems to be responsible of TIC immortalization and, subsequently, tumor progression.

### Conclusions

GBM research is being conducted worldwide at a remarkable pace, with some of the more recent promising studies focused on identification of aberrant genetic events and signalling pathways, tumor stem cell identification and characterization, modulation of tumor immunological responses, combination therapies, and understanding of the rare long-term survivors. Identification of additional indicators will enable better patients’ stratification and individualization of treatment, is needed to more accurately determine the patient’s prognosis and to identify novel therapeutic approaches that can optimize the patient’s outcome. A growing body of knowledge suggest a potential role of telomere length in different tumors. Nevertheless, even if its clinical use its not completely established, a number of studies demonstrated that it can be helpful to patients stratification, to provide useful information about patients prognosis and, in some case, to suggest new therapeutic strategies in cancer diseases. The present review shows that the potential clinical use of telomere length information for the prognosis of GBM has been recognized and continues to be validated. Although not entirely consistent in the type of telomere alteration, i.e., attrition vs. elongation, and unclear on the underlying mechanisms, multiple studies in brain tumors have shown that telomere dysfunctions are associated with parameters of clinical outcome in patients with GBMs. A possible explanation for these interesting discrepancies in brain tumors is the fact that different expression and/or altered regulation of telomerase expression in tumor cells may reflect the underlying biology of telomere maintenance and its dysfunction over time. In telomerase positive tumor cells, telomere length is balanced by telomere shortening due to cell division and telomere elongation by telomerase. In mortal cells, telomeres shorten during proliferation and hence can be considered as a marker for the replicative capacity of cells in vitro. At crisis, the telomeres are at critical length, and the integrity of the chromosomes declines with every subsequent cell division. At this point, telomere length is maintained by telomerase activity that can be influenced in different ways and by various factors. This capacity keeps tumour cells proliferating and growing by the stabilization of their telomeres which is essential to maintain the unlimited dividing potential and to escape ‘crisis’. (8, 34, 71). Therefore, understanding the context and mechanisms by which telomeres length contribute to cancer development was the next logical research step and may represent an interesting research field in order to elucidate GBMs biology. Moving toward the study of molecular mechanisms controlling telomere length, included telomerase and ALT, will not only provide insight into the complex etiology of cancer but also promises to provide novel targets for cancer therapy.

## Figures and Tables

**FIGURE. 1:**
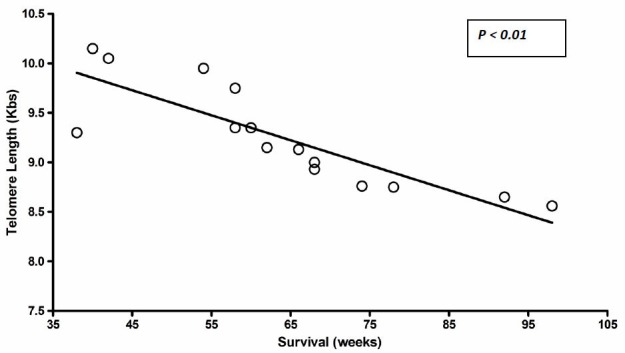
Graph showing inverse correlation between telomere length and survival.

**TABLE 1.** ***Summary of demographic, clinical, and molecular data of 15 patients with Glioblastoma multiforme and controls***

**Patients**	**Age (yr)/sex**	**Localization**	**Duration of symptoms (week)**	**KPS score**	**Survival (week)**	**Telomere length (kb)**
**1**	57/F	R-FP	6	90	42	9.30
**2**	62/M	R-FP	7	100	40	10,15
**3**	68/M	L-O	3	100	38	10,05
**4**	64/F	R-P	7	90	54	9,95
**5**	68/M	L-PT	5	100	58	9,75
**6**	67/F	R-FT	11	70	58	9,35
**7**	65/M	R-O	10	70	60	9,35
**8**	69/M	L-FP	8	80	62	9,15
**9**	73/F	R-PO	4	90	66	9,13
**10**	71/M	R-TP	1	90	68	9
**11**	76/M	R-F	3	90	68	8,93
**12**	68/M	R-T	3	90	74	8,76
**13**	67/F	L-FT	2	90	78	8,75
**14**	62/M	R-F	8	90	92	8,65
**15**	63/F	L-F	5	90	98	8,56
**N.B.T.**	-	-	-	-	-	10.93

Abbreviations used: M: male; F: female; L: left; R: right; F: frontal; P: parietal; T: temporal; O: occipital; KPS: Karnofsky Performance Scale; kb: Kilobase; N.B.T.: normal brain Tissue.

**TABLE 2:** SUMMARY OF RELEVANT FINDINGS FOR TELOMERE LENGTH IN GBMs

**Cases**	**Controls**	**Main findings**	**References**
60 BT	PPBL	Variable length, 41.7% longer, 21.7% shorter, 36.7% maintenance	*Nürnberg P et al. 1993*
12	NBT	Shorter TRFs than controls	Liu J et al. 1996*
4	Telomerase negative GBM, NBT and PPBL	Shorter TRFs in telomerase positive GBMs compared to controls.	*Le S et al. 1998*
77	ALT-negative GBMs	Longer TRFs (more than 17 kb) and significantly longer median survival in ALT-positive tumors than ALT-negative ones.	*Hakin-Smith V et al. 2003*
11	NBMT	Significantly shorter TRFs in high-grade gliomas.	*Maes L et al. 2007*
47	Gliomas without telomerase activity and NBT	Shorter TRFs in tumors with telomerase activity than controls. Within the normal range in those without telomerase activity. 80% of the progression GBMs exhibited reduced mean TRF length (7.747kbp) compared with NBT and origin tumors. In contrast, 56,7% of de novo GBMs showed mean TRF lengths compatible with normal values (9.4–13.2kbp) but the mean TRF length was significantly reduced in tumors with telomerase activity (8.266±0.293) compared with that in the tumors without telomerase activity (11.384±0.922) (P<0.05).	*Hiraga S et al. 1998*
42	NBT	Shorter TRFs than controls. The overall difference between primary and secondary GBM telomere length was not statistically significant	*Harada K et al. 2000*
2 TICs lines	BTC and NTSC	Shorter TRFs than controls	*Castelo-Branco P et al. 2011*
1 TICs line	NBT	Shorter TRFs than controls	*Marian CO et al. 2010*
8	CBTL and PNETs	Longer mean TRFs (8.3 ± 0.24 kb; range 7.2–8.9 kb) than PNETs but equal compared with the mean CBTL TRFs (8.5 ± 0.29 kb)	*Rahman R et al. 2010*
15	NBT	Shorter TRFs than controls. Inverse correlation between telomere length and patients’ survival	*Current series*

BT = Brain Tumors; PPBL = Patients’ Peripheral Blood Leukocytes; Normal Brain Tissue = NBT; TRFs = Telomere Restriction Fragments; GBM = Glioblastoma Multiforme; ALT = Alternative Lengthening of Telomeres; NBMT = Normal Brain and Meningeal Tissue; BTC = Bulk Tumor Cells; TIC= Tumor initiating cell; NTSC = Normal Tissue Stem Cells; CBTL = Constitutional Blood Telomere Length; PNETs = Primitive Neuroectodermal Tumors;

* = data collected only from the abstract
